# Glycosylation-Based Serum Biomarkers for Cancer Diagnostics and Prognostics

**DOI:** 10.1155/2015/490531

**Published:** 2015-10-05

**Authors:** Alan Kirwan, Marta Utratna, Michael E. O'Dwyer, Lokesh Joshi, Michelle Kilcoyne

**Affiliations:** ^1^Glycoscience Group, National Centre for Biomedical Engineering Science, National University of Ireland Galway, Galway, Ireland; ^2^Department of Hematology, National University of Ireland Galway, Galway, Ireland; ^3^Carbohydrate Signalling Group, Microbiology, School of Natural Sciences, National University of Ireland Galway, Galway, Ireland

## Abstract

Cancer is the second most common cause of death in developed countries with approximately 14 million newly diagnosed individuals and over 6 million cancer-related deaths in 2012. Many cancers are discovered at a more advanced stage but better survival rates are correlated with earlier detection. Current clinically approved cancer biomarkers are most effective when applied to patients with widespread cancer. Single biomarkers with satisfactory sensitivity and specificity have not been identified for the most common cancers and some biomarkers are ineffective for the detection of early stage cancers. Thus, novel biomarkers with better diagnostic and prognostic performance are required. Aberrant protein glycosylation is well known hallmark of cancer and represents a promising source of potential biomarkers. Glycoproteins enter circulation from tissues or blood cells through active secretion or leakage and patient serum is an attractive option as a source for biomarkers from a clinical and diagnostic perspective. A plethora of technical approaches have been developed to address the challenges of glycosylation structure detection and determination. This review summarises currently utilised glycoprotein biomarkers and novel glycosylation-based biomarkers from the serum glycoproteome under investigation as cancer diagnostics and for monitoring and prognostics and includes details of recent high throughput and other emerging glycoanalytical techniques.

## 1. Introduction

**Figure 1:**
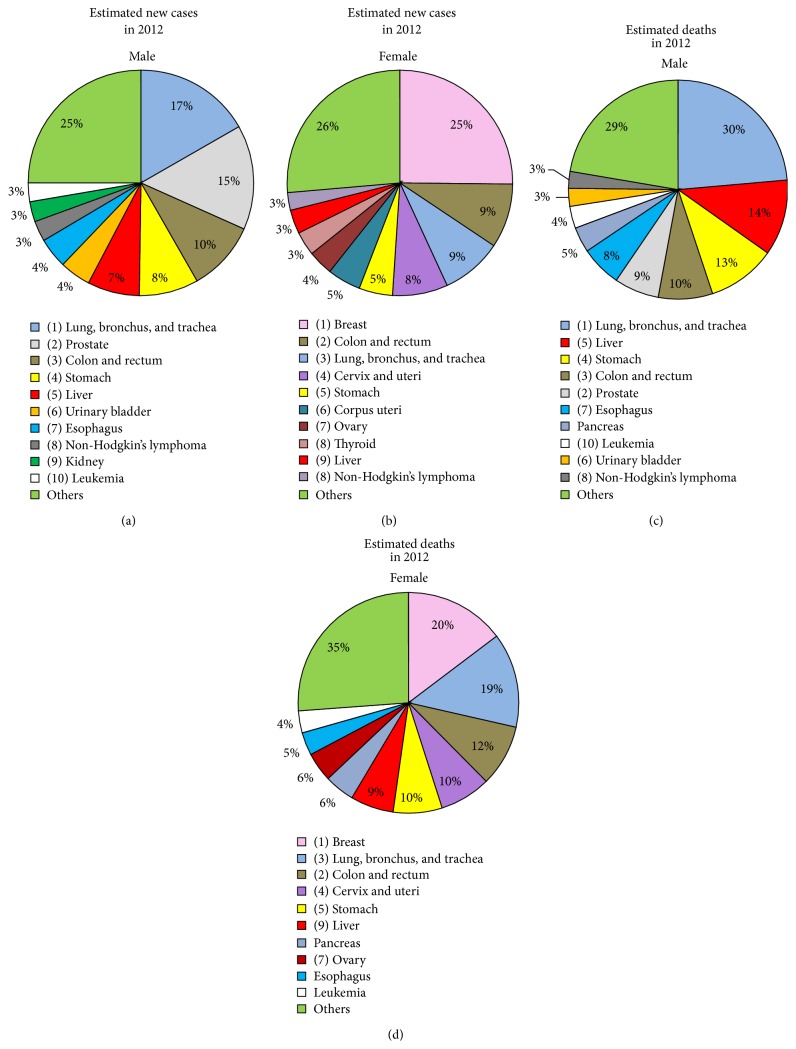
Global cancer statistics. Based on data for 2012 from Torre et al., 2015 [1]. (a) and (b) depict the top 10 most frequently diagnosed types of cancer as a percentage of all detected ones. (c) and (d) represent the top 10 causes of death with each type as a percentage of all cancer-related deaths.

Cancer is the second most common cause of death in developed countries. According to a survey of worldwide cancer rates, there were approximately 14 million newly diagnosed cases and estimated 6,234,000 cancer-related deaths in 2012 [1]. The most commonly diagnosed and leading causes of cancer-related deaths worldwide are malignancies of the lung, bronchus, and trachea in males and breast cancers in females (Figure 1).

Due to a lack of early symptoms and a hesitation to seek medical investigation, many cancer cases are discovered late, when the disease is at a relatively advanced stage. Survival rate is strongly correlated with the stage at which the disease is diagnosed. The early detection of the disease and the development of minimally invasive screening methods that have wide patient acceptability is the most promising approach for improving the long-term survival of cancer patients.

Recent advances in molecular biology tools and computational methods have enabled the identification of novel cancer biomarkers. Biomarkers are currently used as a complementary strategy to imaging or histopathology techniques and aim to provide minimally invasive and source-effective information which can be prognostic and predictive [2].

The current clinically approved cancer biomarkers have greatest value when applied to patients with widespread cancer. However, despite years of effort and a plethora of publications suggesting novel screening tools, single biomarkers with satisfactory sensitivity (ability to detect individuals with the disease) and specificity (ability to distinguish individuals with the disease from those that are either normal or have some other condition) have not been identified for the most common cancers [3]. This is possibly due to the molecular heterogeneity of tumours from patient to patient and the fact that an individual organ can contain a tumour of several stages in the same tissue [4]. Moreover, the majority of cancer biomarkers are elevated in benign diseases, and some biomarkers are undetectable in early stage cancers. However, in most cases extremely abnormal biomarker concentrations correlate to a poor prognosis and inform clinicians that a more aggressive treatment method is required [3]. Thus, despite their limitations, a variety of biomarkers are routinely used in clinical laboratories (Table 1) [5]. Increasing clinical technical capabilities and better characterization of existing biomarkers might contribute to the introduction of multimarker combinations with better diagnostic, monitoring, and prognostic performance and to the discovery of new candidate biomarkers.

Aberrant glycosylation of proteins is a well-known hallmark of cancer and represents a valuable source of information [6, 7]. However, in contrast to proteins and nucleic acids, biosynthesis of oligosaccharides in mammals is not template driven [8]. The structural complexity of carbohydrates underpins their wide range of biological roles and involvement in many cell-cell and cell-matrix interactions related to cancer through modulation of adhesion and cell trafficking [9]. Interestingly, the majority of the human serum proteome is made up of glycoproteins [10]. Proteins enter the circulatory system from tissues or blood cells through active secretion or leakage, including necrotic and apoptotic processes. Thus, carbohydrate structures of great complexity fluctuate in response to multiple stimuli reflecting the physiological and pathological state of the organism. Serum, with its ease of accessibility from the peripheral blood and reduced risk to the patient due to the minimally invasive nature of harvesting, is an attractive option from a clinical and diagnostic perspective [2].

Many technical approaches have been undertaken to describe glycosylation changes associated with cellular conditions and to address the challenges of carbohydrate structure detection and determination [11–16]. In many cases, specific cancer-associated carbohydrate alterations (reviewed elsewhere [6, 17]) can be detected using the separation of oligosaccharides released from glycoproteins by hydrophilic-interaction chromatography (HILIC) high performance liquid chromatography (HPLC), capillary electrophoresis (CE), and mass spectrometry (MS). Monoclonal antibodies and lectins, carbohydrate-binding proteins which are highly specific for various carbohydrate moieties [18], are also commonly employed for the detection of abnormal structures and the proportion of alterations can be quantified [14, 19–21].

The aim of this review is to summarise the current status and the potential for contribution of the serum glycoproteome to cancer diagnostics, monitoring, and prognostics. Glycosylation-based cancer biomarkers which have crossed the boundary from the laboratory into routine clinical use (Table 1) and those which are under development (Table 2), together with the most recent advantages of high throughput (HTP) and other emerging analytical techniques, are described.

## 2. Clinically Approved Biomarkers

### 2.1. *α*-Fetoprotein (AFP)

The presence of *α*-fetoprotein (AFP), a glycoprotein of approximately 70 kDa, was initially reported in the serum of the human fetus in 1956 [22]. AFP is mainly produced by the yolk sac and the fetal liver, reaching its maximum concentration of 3–5 × 10^6^ *μ*g/L at the end of the first trimester [23]. The concentration of AFP in fetal serum decreases to approximately 1–20 × 10^5^ *μ*g/L at term and rapidly declines after birth to adult reference values (0.5–15 *μ*g/L) reached at 2 years of age. Elevated concentrations of AFP also appear in maternal serum during pregnancy and peak at about weeks 30–32 of gestation (200–300 *μ*g/L). Under certain pathological conditions, the expression of AFP is elevated and high serum concentrations are usually an indication of underlying diseases, including hepatocellular carcinoma (HCC), pancreatic and gastrointestinal carcinomas, germ cell tumours of the testis, and brain tumours [24]. Despite its low specificity for individual cancer types, AFP is the best-studied serological biomarker for HCC which is the most common type of liver cancer. Liver cancer is the fifth most frequent type of cancer diagnosed in males worldwide and the second most common in terms of number of cancer-related deaths in males (Figure 1). In a systematic review which evaluated AFP concentrations at all stages of HCC [25], sensitivities of 41–65% and specificities of 80–94% were reported for a cut-off of 20 ng/mL. AFP concentrations are correlated with increased HCC tumour size but have poor sensitivity at early stages, which is insufficient for early detection of cancer [26]. However, a sensitivity of 66% was reported for early stage HCC using a lower cut-off of 10.9 ng/mL [27].

**Figure 2:**
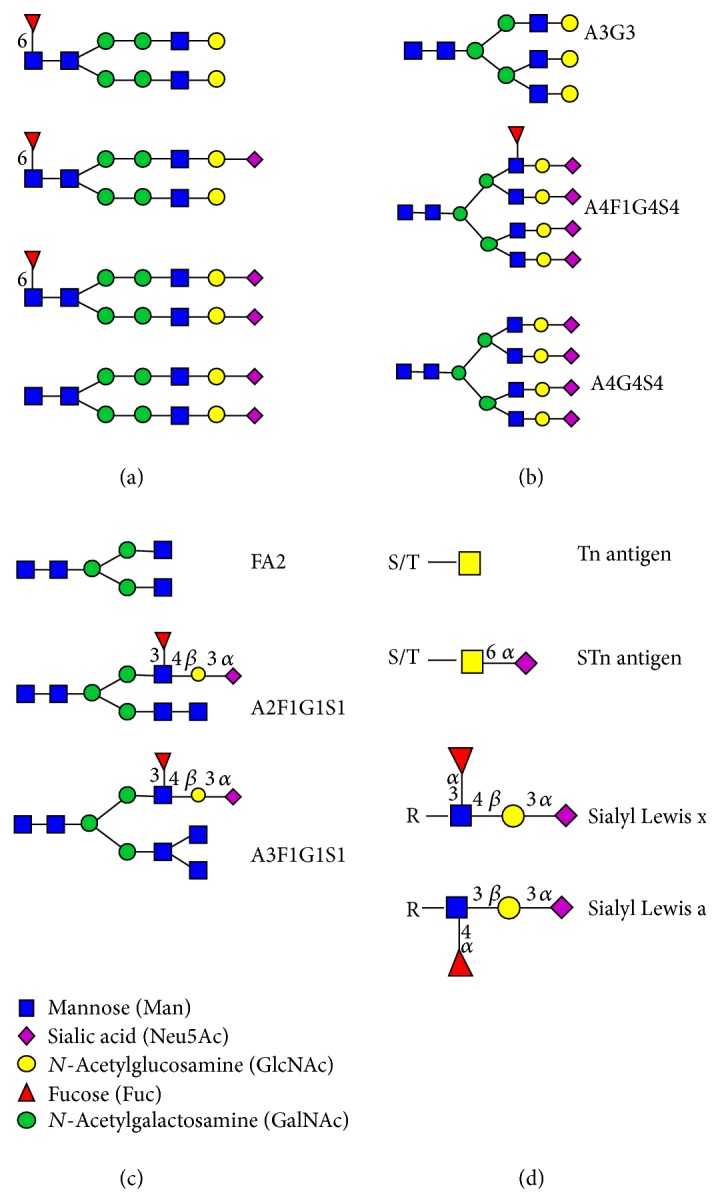
Altered carbohydrate structures expressed in various cancers. (a) *N*-linked oligosaccharides expressed on AFP in HCC patients, the majority of which have core fucosylation based on Johnson et al. [160]. (b) *N*-linked oligosaccharide structures which change in abundance as the cancer progresses according to Saldova et al. [41]. (c) *N*-linked oligosaccharide structures that are upregulated in lymph node metastasis positive breast cancers based on Pierce et al. [159]. (d) Tumour associated carbohydrate structures.

AFP has a single* N*-linked oligosaccharide with a biantennary complex-type structure which has altered terminal sialylation and core fucosylation during cancer (Figure 2). This fucosylation is detectable by the lectin* Lens culinaris* agglutinin (LCA) and increased fucosylation can be correlated with HCC progression [28]. Due to the limitation of AFP concentration for early detection of HCC, the proportion of the LCA-reactive fraction of AFP (AFP-L3) compared to total AFP has been proposed as an improved biomarker [12, 29]. With a 10% cut-off for AFP-L3/AFP, a specificity of 90% and sensitivity of 60% for this biomarker were achieved for all stages of HCC, for those patients with AFP concentrations exceeding 10 ng/mL, including the early disease stages. The United States (U.S.) Food and Drug Administration (FDA) approved a laboratory test for AFP-L3 in 2006 for determining the risk of developing liver cancer [20]. The development of a highly sensitive assay for AFP-L3 enabled measurement in individuals with AFP concentrations as low as 2 ng/mL and the accuracy of this biomarker is under further investigation [30, 31]. The activity of *α*-(1, 6)-fucosyltransferase was also correlated with HCC progression [32]. The addition of measuring the enzymatic activity of *α*-fucosidase, which specifically removes the fucose residue from the* N*-linked oligosaccharide of AFP-L3, can further increase the specificity and sensitivity for the early detection of primary HCC [33]. Coupling the use of the AFP cut-off concentration of 20 ng/mL to classify patients as AFP-positive or AFP-negative [21] with the ratio of fucosylated paraoxonase 1 to paraoxonase can be used to distinguish between HCC and liver cirrhosis (LC) with a sensitivity of 90% and a specificity of 75% in AFP-negative patients. These results were confirmed in a small cohort of patients (20 HCC, 20 LC) in which 17 patients were correctly diagnosed with HCC, providing support for the use of multiple biomarkers as a means of diagnosing early stage cancers [21].

### 2.2. Prostate-Specific Antigen (PSA)

Prostate-specific antigen (PSA), also known as gamma-seminoprotein, kallikrein-3, and KLK3, has been widely used to screen for prostate cancer in men. Prostate cancer is the second most commonly diagnosed cancer and the fifth leading cause of cancer-related deaths in men (Figure 1). PSA is member of the kallikrein family of peptidases and is secreted by the prostate epithelium and periurethral glands. It is a 28.4 kDa glycoprotein with one* N*-linked glycosylation site and is further subcategorized into glycosylated (gp28, gp22, gp18, and gp12) or nonglycosylated (p26-full length nonglycosylated PSA, p20, p16, p10, and p6) peptides [34]. The function of PSA is to liquefy semen in the seminal coagulum to enable sperm to swim in the ejaculate.

Disruption of the prostatic epithelium in inflammation and prostate disorders, including benign prostatic hyperplasia (BPH) and prostate cancer, causes diffusion of PSA into the tissue around the epithelium and leads to elevated concentrations of circulating PSA in these conditions. PSA is present in small quantities in the serum of men with healthy prostates (up to 2.5 ng/mL before their 40 s and around 6.5 ng/mL after 70 years of age) but concentrations above 4 ng/mL are considered indicative of prostate cancer or BPH [35]. PSA as a diagnostic by itself currently has a low specificity and has led to extensive overdiagnosis, overtreatment, and potential harm, especially from unnecessary biopsies. However, serum PSA screening in conjunction with a digital rectal exam (DRE) and Gleason scoring of prostate biopsy samples has been approved by the FDA for the early detection of prostate cancer [36, 37]. Recent approaches for improving the specificity and sensitivity of the serum PSA test include research into the individual molecular forms of PSA (proPSA, benign PSA, and intact PSA), kallikreins other than PSA, calculating the proportion of total PSA complexed with *α*1-chymotrypsin and *α*2-macroglobulin (tPSA) compared to free PSA (fPSA), comparing PSA with other markers such as prostate cancer antigen 3 (PCA3) [38] and examining PSA modifications such as glycosylation [39–41].

Several studies have reported altered fucosylation and sialylation in PSA and other proteins isolated from the serum of prostate cancer patients [42, 43]. Serum PSA contains an additional *α*-(2,3)-linked sialic acid to the terminal galactose residue on *N*-linked oligosaccharides in prostate cancer when compared to healthy individuals [39, 44] (Figure 2). The binding of prostate cancer-associated PSA to the *α*-(2,3)-linked sialic acid-recognizing lectin* Maackia amurensis* agglutinin (MAA) was more intense compared to PSA from a healthy individual [13]. Analysis of PSA by 2D electrophoresis identified five PSA glycoforms (F1, F2, F3, F4, and F5) in prostate cancer and BPH sera [40]. The F5 glycoform was nonglycosylated and the F4 glycoform had a lower degree of sialylation compared to the F1–F3 glycoforms. The *N*-linked oligosaccharides on the most abundant PSA glycoform F3 had a greater proportion of *α*-(2,3)-linked sialic acid and a decrease in core fucosylation in prostate cancer (Figure 2). The relative percentage of F3 (%F3) compared to all glycoforms (F1–F5) negatively correlated with the stage of prostate cancer while the relative percentages of the F4 (%F4) glycoform, which contained monosialylated *N*-linked oligosaccharides (Figure 2), were increased in prostate cancer patients [40].

Li et al. [45] showed that fucosylated PSA had better predictive power to differentiate between aggressive and nonaggressive forms of prostate cancer compared to total PSA. Yoneyama et al. [46] used a magnetic microbead-based immunoassay and a free serum PSA glycoform that terminates in *α*-(2,3)-linked sialic acid to develop a more sensitive diagnostic PSA assay. The novel assay has a sensitivity of 90.2% and a specificity of 64.2% when used on a cohort of patients with (*n* = 138) and without (*n* = 176) prostate cancer. This method was more sensitive and accurate than either PSA alone or percentage of fPSA in diagnosing prostate cancer in these patients.

Routine differentiation between prostate cancer and BPH is far from clear-cut and on-going research concentrates on the altered microheterogeneity of each PSA glycoform to distinguish between the two conditions [47, 48].

### 2.3. MUC16 (CA125)

MUC16, initially named cancer antigen 125 (CA125), was first described as a biomarker in a screen of monoclonal antibodies developed against the OVCA433 ovarian cancer cell line [49]. MUC16 is a membrane-spanning mucin and the largest mucin known to date. It has a molecular mass as high as 2 × 10^6^ Da [50, 51]. MUC16 is expressed by the various normal epithelial cells of the human body, including bronchial, endometrial, ovarian, and corneal. MUC16 protects the cells and sheds its extracellular portion into the bloodstream. Soon after its discovery, MUC16 was established as a serum biomarker for diagnosing and monitoring stability or progression in ovarian cancer [52]. However, observations of other conditions including nongynecological cancers and benign conditions such as endometriosis, as well as individuals during menstruation and pregnancy, reported elevated MUC16 serum concentrations [53]. Despite being nonspecific and unreliable for diagnosing early stage ovarian cancer, monitoring serum MUC16 together with ultrasonography is a standard procedure for detection of ovarian malignancies [54].

Several studies have reported attempts to use MUC16 glycoforms to discriminate between endometriosis and ovarian cancer and to evaluate the clinical stage, cytological grade, and histological type of ovarian cancer [55–57]. Varying concentrations of sialyl-Tn antigen (STn, Neu5Ac-*α*-(2,6)-GalNAc-*α*-*O*-Ser/Thr) were expressed in MUC16-enriched fractions from the peritoneal fluid of patients with endometriosis and ovarian cancer [55]. A lectin microarray analysis of selected carbohydrate structures, including STn and Tn (GalNAc-*α*-*O*-Ser/Thr) (Figure 2), on MUC16 and MUC1 (CA15-3) was able to distinguish benign ovarian neoplasms from invasive epithelial ovarian/tubule cancer with a specificity of 61.1% and 90% sensitivity [56]. This HTP method is a promising approach for differential diagnosis and requires further investigation in other cancers.

### 2.4. Human Epididymis Protein 4 (HE4)

Human epididymis protein 4 (HE4, also known as WFDC2) was first identified in differential cDNA screening of human epididymal tissue [58, 59]. HE4 is a small (23–27 kDa), secretory protein with hydrophobic amino acids at the N-terminus consistent with a signal peptide which cleaves to yield a mature secretory polypeptide with a consensus site for *N*-linked glycosylation at amino acid position 15. HE4 contains two whey acidic protein (WAP) domains characterized by a four-disulfide core arrangement of 50 amino acids, including eight cysteines. Based on gene expression data, the HE4 gene is one of the most frequently upregulated genes in epithelial ovarian carcinomas [60, 61]. HE4 has also been shown to be expressed and secreted as a glycoprotein by ovarian carcinoma cells [62]. Moreover, HE4 expression is lower than MUC16 in benign gynecological conditions and low-malignant potential tumours and HE4 is found in a fraction of endometrial and ovarian cancers which are deficient for MUC16 expression.

In June 2008, the HE4 enzyme immunoassay (EIA) test kit (Fujirebio Diagnostics, Sweden) and, in March 2010, the ARCHITECT HE4 automated version (Abbott Diagnostics, UK) were approved by the FDA as substantially equivalent to a MUC16 assay for ovarian cancer. The HE4 EIA is a solid-phase, noncompetitive immunoassay based on the direct sandwich technique, which measures concentrations between 15 pM and 900 pM [63]. The most recent review of the performance of the HE4 and MUC16 in multiple studies concluded that HE4 exhibits a significantly higher specificity than MUC16 (93%* versus* 78%, resp.) and outperforms MUC16 in identifying patients with early stage ovarian cancer [64]. In September 2011, the FDA approved the combination of the HE4 test with the MUC16 test in the Risk of Ovarian Malignancy Algorithm (ROMA) test, to determine the likelihood of finding malignancy at surgery in premenopausal or postmenopausal women presenting with an ovarian adnexal mass [65, 66]. A study involving 349 female patients with pelvic masses and with different menopausal status confirmed that the ROMA test outperforms the individual biomarkers in their ability to detect both early and late stage ovarian cancers, and this reached statistical significance in postmenopausal women [67].

Despite the fact that HE4 was shown to be glycosylated [62, 68], there has only been limited studies addressing the role of glycosylation for HE4 function [69] and none on the diagnostic or prognostic capability of different glycoforms. Further studies of HE4 glycoforms may lead to insights in to the occurrence, development, or migration of cancerous cells and facilitate early diagnosis or improve the therapeutic options in ovarian cancer.

### 2.5. MUC1 (CA15-3/CA27.29)

MUC1, also known as cancer antigen 15-3 (CA15-3), MAM6, milk mucin antigen, and CA27.29 [70], is a transmembrane mucin expressed by most glandular epithelial cells as a high molecular mass glycoprotein which is heavily substituted with* O*-linked oligosaccharides. It was first identified in human milk, where it is shed from lactating mammary epithelial cells which surround the fat globules [71, 72]. MUC1 was identified on the surface of many types of cancer cells, for example, breast and ovarian, lung, pancreatic, and prostate cancers [70]. It is shed into the blood stream where it can be found in the serum of cancer patients in considerable amounts by certain therapeutic antibodies. To date, multiple monoclonal antibodies, recognizing different portions of the molecule, have been developed against the mucinous antigens of MUC1 [73–75]. Thus, in many publications, the terms CA15-3- and CA27.29-targeting epitopes of MUC1 protein are used interchangeably. Despite the lack of specificity, MUC1-directed assays in combination with other serum biomarkers are routinely used in the complex diagnosis of breast cancer [76]. The anti-CA27.29 monoclonal antibody developed against one of the MUC-1-associated epitopes binds to an eight-amino-acid sequence that partially overlaps the antigen binding site for the DF3 antibody [77]. Thus, it provides comparable results to the first results reported in MUC1 tests assessed by anti-CA15-3 radioimmunoassay [74, 77]. However, serum monitoring with the CA27.29 antibody cannot distinguish stage I from stage II patients and CA27.29 monitoring is primarily used in metastatic breast cancer to detect treatment failure in the absence of readily measurable disease [74].

The altered glycosylation of serum MUC1 in breast cancer is another possibility for the early diagnosis of breast cancer. MUC1 in breast malignancies is more heavily glycosylated in comparison to MUC1 from a healthy tissue and MUC1 peptide fragments bearing aberrant* O*-linked oligosaccharides are secreted from epithelial cell surfaces to serum [78]. The* O*-linked oligosaccharides on the MUC1 shed in to serum of an advanced breast cancer (ABC) patients were analysed by HPLC [79]. Mucin type core 1* O*-linked oligosaccharide structures dominated (83%) over core 2 structures (17%) and the majority of structures had high levels of sialylation. Additionally, truncated structures of MUC1 are observed on tumor cells with short, often prematurely sialylated side chains of oligosaccharides, including the Thomsen-Friedenreich antigen (T antigen), its precursor (Tn antigen), and their respective sialylated derivatives STn and *α*-(2,6)-sialylated T antigen (Figure 2). Because MUC1 antigen is abundantly expressed and aberrantly glycosylated in carcinomas, the tumor associated glycopeptides and epitopes which are masked in normal cells are considered an attractive target in cancer immunotherapy and immunodiagnostics and have been the subject of intensive research efforts [80–82].

### 2.6. Human Epidermal Growth Factor Receptor 2 (HER2)

The human epidermal growth factor receptor 2 (HER2) is encoded by the ERBB2 gene and is also known as cluster of differentiation 340 (CD340) or protooncogene Neu. It is a 185 kDa glycoprotein consisting of three domains; a 105 kDa extracellular domain (ECD), a transmembrane lipophilic segment, and an intracellular domain with tyrosine kinase activity. The ECD portion can be released by cleavage from the HER2 receptor and shed into serum [83]. Overexpression of HER2 is observed in 20–30% of breast cancers, resulting in an aggressive tumour phenotype, reduced survival, and possible treatment eligibility with the monoclonal antibody trastuzumab or other therapies targeted against the HER2 receptor protein [84, 85]. The prognostic value of HER2 ECD combined with MUC1 in early breast cancer was shown to be valuable in identifying high-risk breast cancer patients. These two independent indicators of a worse disease-free survival are used to identify patients in need of more aggressive therapies and intensified surveillance [86]. HER2 ECD has also been shown to be a potential diagnostic and prognostic biomarker in HER2-positive gastric cancer. Not only was there a direct correlation between serum and tumour HER2 concentration, there was also a correlation between serum HER2 concentration and patient responses to chemotherapy [87].

Although the ECD of HER2 contains several potential *N*-linked glycosylation sites, studies of its glycoforms have been limited to an examination of how HER2 glycosylation affected the specificities of a panel of anti-HER2 antibodies [88]. HER2 oligosaccharide structures have not yet been elucidated.

### 2.7. Carcinoembryonic Antigen (CEA)

Carcinoembryonic antigen (CEA) has molecular weight of approximately 180 kDa, belongs to the immunoglobulin superfamily, and is a glycosylphosphatidylinositol-anchored cell surface glycoprotein. CEA is normally produced by mucosal cells in gastrointestinal tissue during fetal development and the expression decreases before birth, with the highest concentrations in the second trimester 80–100 ng/mL in amniotic fluid at week 19, reducing to 50 ng/mL at full term [89]. CEA is not elevated in maternal serum during pregnancy since it does not cross the placenta and is present only at very low concentrations in healthy adult serum of both genders (less than 2.5 ng/mL). However, CEA serum concentrations are elevated for heavy smokers, who express up to 5 ng/mL, and under certain pathological conditions, including colorectal, gastric, pancreatic, nonsmall cell lung, and breast carcinomas [90]. CEA is the primary biomarker used for the staging of colorectal carcinoma and monitoring the recurrence or spread of colon cancer after surgical resection, as rising concentrations of CEA precede other clinical indicators by several months [91, 92].

*N*-linked oligosaccharides account for more than 50% of the molecular mass of CEA and it is hypothesized that the reduction in mass of human colonic CEA to 170 kDa is as a result of alterations in glycosylation [93, 94]. CEA expressed by CD44-double knockdown LS174T colon carcinoma cells is more densely substituted with sialylated and fucosylated epitopes than CEA on wild-type LS174T cells [95]. The avidity of the altered glycoforms of CEA for selectins was increased when compared to glycoforms from the wild-type cells, which may contribute to metastatic dissemination [95]. However, further studies are required for CEA glycosylation and the role of this glycosylation and to investigate whether these potentially altered glycoforms can enhance the diagnostic ability of CEA.

### 2.8. Carbohydrate Antigen (CA19-9)

Carbohydrate antigen 19-9 (CA19-9), or cancer antigen 19-9, is the sialyl Le^a^ (sLe^a^) blood group structure which is recognised by the antibody N-19-9 [96] (Figure 2). CA19-9 is used primarily in combination with other biomarkers (e.g., CEA) for the monitoring and management of pancreatic cancer [90]. CA19-9 is also currently recognised as one of the most common tumour markers for colorectal, gastric, and hepatocellular cancer [97]. The latter three types of cancer contribute to 28% and 16% of cancer-associated deaths in males and females, respectively (Figure 1). The biggest disadvantage of using the CA19-9 testing is that sLe^a^ structure is neither exclusively expressed for a specific tumour type nor is it expressed in cancer only, but it is expressed at a lower concentration in tissue and serum of healthy individuals of appropriate blood types. Additionally, patients who are genotypically negative for the Le^a^ antigen cannot produce CA19-9, even when affected by cancer [98].

Increased expression of CA19-9 is used to indicate the presence of pancreatic cancer before any evidence of disease is obtained with other methods [99] and strictly correlates with the clinical response after pancreatectomy. Thus, it is used for the monitoring of disease recurrence [100]. Similarly, CA19-9 testing in combination with other biomarkers was recommended in multiple studies for estimating the relapse of gastric carcinoma after surgery [101, 102]. Recently, CA19-9 has been used as a prognostic biomarker for HCC and postoperative cholangiocarcinoma patients. In HCC, patients serum concentrations in excess of 100 U/mL independently predicts poorer overall survival while, in cholangiocarcinoma patients, serum CA19-9 concentrations in excess of 150 U/mL were associated with a worse overall survival [97, 103].

### 2.9. Thyroglobulin (Tg)

Thyroglobulin (Tg) is a 660 kDa dimeric glycoprotein with 20 potential *N*-linked glycosylation sites, of which 16 sites were shown to be glycosylated in the mature protein [104]. Tg is produced by the follicular cells of the thyroid and is used by the thyroid gland as a substrate for the synthesis of thyroxine and triiodothyronine and for the storage of the inactive forms of thyroid hormone and iodine. Serum Tg concentration is a biomarker for monitoring postoperative thyroid cancer recurrence [105, 106]. However, the usefulness of preoperative Tg measurements (partly related to difficulties with antibody interference and nonspecific recognition) remains unclear [107–110]. The glycosylation of Tg is well known and carbohydrate structures correlated with Tg function playing a role in the secretion of Tg, transportation of Tg to cell compartments, iodination, hormone synthesis, and immunoreactivity [104].

Structure elucidation of Tg glycosylation in cancer has not been performed to date but may be useful for thyroid cancer diagnostics. Preliminary studies showed that the interaction of lectin LCA with Tg from thyroid carcinoma was significantly lower than that in normal thyroid tissue and in patients with benign thyroid tumor [111, 112]. The percentage of LCA-reactive Tg could discriminate between benign and malignant lesions [113]. It was also found that the percentage of LCA-reactive Tg was significantly decreased in thyroid carcinoma patients who were positive for lymph node metastasis compared to thyroid carcinoma patients who were negative for lymph node metastasis [112].

## 3. Potential Novel Biomarkers

The translation of biomarkers from discovery to clinical practice is still ongoing for hundreds of potential biomarkers which have been identified and published. The validation process of a putative biomarker requires time, hundreds of specimens, and large cohorts of patients to be shown reproducibly. Examples of promising biomarkers routinely checked in clinical practice but not approved for specific cancer due to low specificity or sensitivity are described below.

### 3.1. Human Chorionic Gonadotropin (hCG)

Human chorionic gonadotropin (hCG) is a heterodimeric glycoprotein hormone produced by the placenta and comprises an *α*-subunit and a *β*-subunit that can vary in glycosylation [114]. The *α*-subunit structure is common to luteinizing hormone, follicle stimulating hormone, and thyroid stimulating hormone while the *β*-subunits of the aforementioned hormones display various degrees of homology with each other, conferring the distinct biological activity of each heterodimer. In addition, two variants of hCG, regular and hyperglycosylated, have independent activities. The regular form maintains the arteries and the vascular supply of the placenta during the full course of pregnancy while the hyperglycosylated hCG (hCG with* O*-linked oligosaccharides) is responsible for embryo implantation during pregnancy [115].

The hyperglycosylated form of hCG is also expressed by several tumours, including male germ cell tumours (GCTs) and choriocarcinomas [114], and has been suggested to play a central role in cancer invasion [116]. High concentrations of hCG are usually indicative of adverse prognosis for cancer progression [114, 117]. More complex carbohydrate structures were reported for cancer-related hCG when compared to hCG expressed during pregnancy [118, 119]. However, the relative proportion of hCG isoforms may vary among healthy and diseased states and false positive hCG results are a major problem in the management of gestational trophoblastic disease and cancer [120]. While hCG is well-known indicator of tumours, it has not been approved for this application by the FDA.

### 3.2. *α*-1-Antitrypsin (A1AT)

A1AT is a 52 kDa serine protease inhibitor with three potential glycosylation sites which is produced mainly by hepatocytes and is upregulated in the serum of lung cancer patients [121–124]. A1AT is present in various different glycoforms which can be used to distinguish between various subtypes of lung cancer and benign pulmonary diseases (BPDs) [124]. The galactosylated A1AT and fucosylated A1AT glycoforms can both distinguish nonsmall cell lung carcinoma (NSCLC) (*n* = 23) from BPD (*n* = 25) with identical degrees of accuracy (AUC = 0.834). Fucosylated A1AT can also efficiently distinguish adenocarcinoma (*n* = 28) from BPD (AUC 0.919). The poly-*N*-acetyllactosamine (polyLacNac) A1AT glycoform can distinguish between small cell lung carcinoma and BPD with a high degree of accuracy (AUC = 0.905) [124]. While the preliminary data is promising, these biomarkers were only examined on 81 patients and need to be investigated in a larger cohort of patients.

### 3.3. Fucosylated Haptoglobin (Fuc-Hpt)

Haptoglobin is a 40 kDa glycoprotein that is produced mainly in the liver and has a low proportion of fucosylation in healthy individuals [125, 126]. Highly fucosylated haptoglobin (Fuc-Hpt) was identified as a potential biomarker in pancreatic cancer upon* Aleuria aurantia* lectin (AAL) blot analysis of the serum of pancreatic cancer patients [127]. Fuc-Hpt has also been shown to be upregulated in the serum of pancreatic cancer patients, with increased branching and fucosylation of the antennae of the *N*-linked oligosaccharides on the beta chain of Hpt [128]. Fuc-Hpt of pancreatic cancer patients had more intense binding to AAL compared to the healthy controls [125]. The concentrations of Fuc-Hpt in 300 pancreatic cancer patients and 315 healthy volunteers were analysed using lectin-based ELISAs. Fuc-Hpt concentrations were significantly higher in the pancreatic cancer patients (*P* < 0.01) and the ELISA had an AUC of 0.91, a sensitivity of 85.1%, and a specificity of 82.3% [129]. Fuc-Hpt was also elevated in certain colorectal cancer patients, in relation to the proximity of the tumour to the liver and distance metastasis. When Fuc-Hpt was combined with CEA, it was shown that it had the potential to be a novel prognostic marker in colorectal cancer [125, 130]. Fuc-Hpt could also be a potential prognostic biomarker in prostate cancer, as it significantly correlated with Gleason scores and biochemical recurrence after radical prostatectomy. PSA also correlated with overall and progression-free survival and the clinical stage of prostate cancer [131].

### 3.4. YKL-40

YKL-40, also known as chitinase-3-like 1 (CHI3L1) or human cartilage glycoprotein-39, is a 40 kDa secreted glycoprotein with two potential *N*-linked glycosylation sites which has been proposed as a biomarker in a variety of cancers but has not received FDA approval [132]. High serum concentrations of YKL-40 have previously been associated with high risk disease and increased bone destruction [133, 134]. YKL-40 was investigated as a prognostic marker in multiple myeloma (MM) [135]. A study carried out in 230 MM patients showed that age-corrected serum YKL-40 concentration is an independent prognostic biomarker in MM and indicates a quicker progression to the first skeletal related complications (e.g., bone lesions) [135]. The data shown is promising but a larger multicentre clinical trial is required before YKL-40 can be accepted as a prognostic marker in MM.

## 4. Carbohydrates as Potential Serum Biomarkers

Advances in HTP glycoanalytical methods have led to investigation of the carbohydrate structures present on glycoproteins in the serum of cancer patients and healthy controls. Many research groups have evaluated whether the variation in structure and/or abundance of these carbohydrates can distinguish between cancer patients and healthy controls [136–139]. This section presents the most recent publications (from 2010 to the present) on alterations in carbohydrate structures on serum glycoproteins of cancer patients and their potential utility as clinical biomarkers.

### 4.1. Ovarian Cancer

The biomarker currently used to diagnose ovarian cancer, MUC16, can only detect late stage ovarian cancer and cannot distinguish between ovarian cancers and benign ovarian diseases (BOD). Enzyme-released *N*-linked oligosaccharides from the serum of patients with ovarian cancer and BOD were analyzed by MS [138, 140]. MS analysis revealed a panel of *N*-linked oligosaccharides which could accurately distinguish between ovarian cancer and BOD with greater sensitivity (81–84%) and specificity (83%) than MUC16 (sensitivity = 78%) when tested on a small cohort of patients (37 ovarian patients and 23 healthy controls [140] and 20 ovarian cancer patients, 20 BOD patients, and 33 healthy controls [138]). The use of carbohydrates as improved biomarkers for diagnosing ovarian cancer compared to MUC16 is currently being investigated in a clinical trial (NCT00628654, Table 2).

Increased sialylation is a common glycosylation alteration in various cancer types and sialylation has been investigated as a possible cancer biomarker [141–143]. Measuring the alteration in the serum concentrations of both sialic acid and hydroxyproline distinguishes between ovarian cancer and healthy controls [144]. This assay outperformed the MUC16 and HE4 assays in the diagnosis of ovarian cancer [44]. However, this assay was not significantly better than the ROMA test, which currently remains the best method for diagnosing and monitoring ovarian cancer [66].

### 4.2. Gastric Cancer

Gastric cancer is the second most common cause of cancer-related death (Table 1). Studies have shown that the infections with* Helicobacter pylori* which cause gastritis can progress to gastric adenocarcinoma [145–148].* H. pylori* infection is associated with a sixfold increased risk of gastric cancer [149].* H. pylori* infection also causes peptic ulcer disease but, unlike gastritis, it is inversely correlated to gastric cancer. However, there are currently no methods for the early stage detection of gastric cancer and most cases present with advanced or metastatic disease [150]. MS was used to investigate whether alterations in the structure or abundance of* N*-linked oligosaccharides in the serum of gastric cancer patients (*n* = 36) could distinguish them from patients with gastritis (*n* = 18) or duodenal ulcers (*n* = 18) [139]. Gastric cancer patients had altered serum *N*-linked glycosylation when compared to patients with gastritis. Gastric cancer patients showed reductions in high-mannose type *N*-linked oligosaccharides, those with one complex-type antenna and bigalactosylated biantennary structures and increased levels of nongalactosylated biantennary *N*-linked oligosaccharides [139]. Significant differences in *N*-linked oligosaccharides only existed between gastric cancer and gastritis patients. While these results will need to be confirmed in a larger cohort of patients, it does support the use of serum glycosylation as potential diagnostic biomarkers in gastric cancer.

### 4.3. Pancreatic Cancer

Alpha-1-acid glycoprotein (AGP), a 40 kDa acute phase serum glycoprotein with five complex-type *N*-linked oligosaccharides attached to the polypeptide backbone, shows variations in abundance and glycosylation in various different cancers [151]. The structures of the *N*-linked oligosaccharides on AGP from patients with pancreatic cancer (*n* = 6) and patients with chronic pancreatitis (*n* = 2) were analysed using LC-MS to investigate their potential as diagnostic biomarkers [152]. There was an increase in fucosylated triantennary trisialylated and fucosylated tetra-antennary trisialylated *N*-linked oligosaccharides in the pancreatic patients when compared to the pancreatitis patients. The increased abundance of these *N*-linked oligosaccharides also differed between the various stages of pancreatic cancer and could be potentially used as prognostic biomarkers [152]. While the sample size used in this study was too small for a statistical analysis to be carried out, a larger cohort of patients can confirm whether these glycosylation alterations can be used as diagnostic and prognostic markers.

Current clinical interest in AGP is related to its abundance in the serum of cancer patients. The serum concentration of AGP affects the pharmacokinetics and dynamics of the chemotherapeutic drug docetaxel and may predict a patient's reaction to the therapy [153]. The effect of AGP on docetaxel therapy is currently being examined in a large scale clinical trial (Table 2).

Another serum glycoprotein that is abnormally glycosylated in pancreatic cancer is ceruloplasmin. Ceruloplasmin is an acute-phase protein that is produced by the liver and secreted into the plasma. Ceruloplasmin has four *N*-linked glycosylation sites with complex type, bi-, tri-, and tetra-antennary structures, fucosylated and sialylated, containing the sialyl Lewis x (sLe^x^) epitope (Figure 2) [154]. Analysis of the *N*-linked oligosaccharides on ceruloplasmin using MS showed that it had a trend towards higher proportions of sLe^x^ in pancreatic patients (*n* = 20), when compared to healthy controls (*n* = 13) and patients with chronic pancreatitis (*n* = 14) [155]. A larger sample size is required before it can be confirmed whether the trend of higher sLe^x^ expression on ceruloplasmin can be used as biomarker for pancreatic cancer diagnosis and progression.

### 4.4. Colon Cancer

The expression of the cancer-related epitopes sLe^x^ and sLe^a^ on glycoproteins present in the serum of colon cancer patients was analysed using a novel antibody microarray [156]. A panel of five serum glycoproteins were identified that could distinguish between stage 3 and stage 4 colon cancer patients and healthy controls with an AUC of 90%. Although the glycoproteins were not named, they may represent novel biomarkers that could improve the sensitivity of current tests for colorectal cancer.

### 4.5. Oesophageal Cancer

LC-MS was used to determine the site specific alterations in *N*-linked oligosaccharides in oesophageal cancers [137]. This novel method was applied to serum isolated from patients with oesophageal cancer (*n* = 15) and disease-free controls (*n* = 15). The study also included patients with diseases that can develop into oesophageal cancer, for example, high grade dysplasia (*n* = 12) and Barrett's disease (*n* = 7) [137, 157, 158]. Significant alterations in site-specific glycosylation were successfully identified on the serum proteins vitronectin, ceruloplasmin, alpha-2-macroglobulin, and complement factor 1 between oesophageal cancer and control patients. These findings will have to be verified in a larger cohort of patients before any definitive conclusions can be made.

### 4.6. Breast Cancer

Increased sialylation, changes in fucosylation, and higher proportions of sialyl Lewis x were reported in *N*-linked oligosaccharide structures in serum from breast cancer patients [19]. The abundance of sLe^x^ containing *N*-linked oligosaccharides was investigated in the serum from 52 breast cancer patients and 134 patients with benign breast disease using exoglycosidase digestion and HPLC analysis to determine whether it could be used as a diagnostic/prognostic tool. While there was no significant difference in serum glycosylation between early stage breast cancer and benign breast disease, there were differences in serum glycosylation between breast cancer patients with lymph-node positive and lymph-node negative breast cancer. Patients with lymph-node positive breast cancer showed increased proportions of biantennary (FA2) and terminally sialylated* N*-linked oligosaccharides (A3F1G1S1 and A2F1G1S1) containing the sLe^x^ structure in their serum when compared to lymph node-negative patients with early breast cancer [159]. These results need to be confirmed in a larger cohort of patients to verify the prognostic utility of serum glycan analysis in breast cancer.

### 4.7. Prostate Cancer

Analysis of serum glycosylation using HPLC and exoglycosidase digestion showed taht there were differences in fucosylation and sialylation between prostate cancer patients and patients with BPH. Serum form prostate cancer patients had increased core fucosylation, as well as increased expression of *α*-(2,3)-linked sialic acid when compared to serum BPH patients [41]. These alterations in serum glycosylation could also distinguish between different stages of prostate cancer. Triantennary trigalactosylated (A3G3) and tetra-antennary tetrasialylated *N*-linked oligosaccharides with outer arm fucose (A4FS4) (Figure 2) were significantly decreased on serum PSA from patients with a Gleason score of 7 (more aggressive cancer and a higher chance of relapse) compared to a Gleason score of 5. In contrast, tetra-antennary tetrasialylated *N*-linked oligosaccharides (A4S4) (Figure 2) were increased in the serum of PSA patients with a Gleason score of 7. The serum glycome analysis was better than PSA at distinguishing between BPH and prostate cancer and at distinguishing between patients with a Gleason score of 7 and patients with a Gleason score of 5 [41]. While the results of this study are promising, they must be confirmed in a larger cohort of patients.

## 5. Conclusions

Research into cancer-specific alterations in glycosylation of serum glycoproteins has provided a promising source of novel biomarkers. Various groups have reported that altered glycoforms of serum glycoproteins can be used to diagnose and monitor various cancers with greater sensitivity and specificity than the currently used biomarkers [21, 44, 156]. Preliminary data has shown that serum glycome analysis is potentially a very sensitive method of discriminating between cancer and control patients or patients with related benign conditions and can detect cancers at a much earlier stage than the currently used biomarkers [139, 140]. Given the possible diagnostic power of glycoproteins and serum glycome analysis, glycosylation-based biomarkers are currently one of the most promising areas of biomarker discovery.

## Figures and Tables

**Table 1:** List of FDA-approved cancer biomarkers currently used in clinical practice.

Marker	Full name	Cancer types	Detection type	Clinical applications	Year of FDA approval
AFP	*α*-Fetoprotein	Liver	Protein concentrations and core fucosylation (for AFP-L3)	Diagnosis, staging, detecting recurrence, and monitoring therapy	1992/2008

PSA, Pro2PSA	Prostate-specific antigen	Prostate	Protein concentrations	Screening, discriminating cancer from benign disease	1986/1994/ 2012

CA125 (MUC16)	Cancer antigen 125	Ovarian	Protein concentrations	Monitoring therapy, detecting recurrence	1997/2011

HE4 (WFDC2)	Human epididymis protein 4	Ovarian	Protein concentrations	Monitoring therapy, detecting recurrence	2008

OVA1 test (multiple proteins)	*β*-2 Microglobulin + CA 125II (up), apolipoprotein A1 + prealbumin + transferrin (down)	Ovarian	Protein concentrations	Prediction	2009

ROMA test	HE4 + CA125	Ovarian	Protein concentrations	Prediction	2011

CA15-3 (MUC1)	Cancer antigen 15-3	Breast	Sialylated *O*-linked oligosaccharide on MUC1	Monitoring therapy	1997

CA27-29	Cancer antigen 27-29	Breast	MUC1 protein levels	Monitoring therapy	2002

CA19-9	Carbohydrate antigen 19-9 or cancer antigen 19-9	Pancreatic, ovarian	SLe^a^ on mucin glycoproteins and gangliosides	Monitoring therapy	2002

CEA	Carcinoembryonic antigen	Colon, gastric, pancreatic, lung, and breast	Protein concentrations	Monitoring therapy, detecting recurrence	1985

HER2/neu	Human epidermal growth factor receptor 2	Breast	Protein concentrations	Therapy choice	1998

Tg	Thyroglobulin	Thyroid	Protein concentrations	Monitoring therapy	1997

hCG	Human chorionic gonadotropin	Testicular, ovarian	Protein concentrations	Diagnosis, staging, detecting recurrence, and monitoring therapy	Not approved

**Table 2:** Clinical trials using blood/plasma or serum carbohydrate analysis to diagnose and monitor cancer. Information on recent clinical trials (https://clinicaltrials.gov/) that involve analysis of glycosylation-based biomarkers in blood components to monitor and diagnose various cancers. ^*∗*^Status of trials was correct at time of submission (April 2015).

Trial title	Description of trial	Status^*∗*^	Clinicaltrials.gov identifier
Glycoprotein and Glycan in Patients with Stage I, Stage II, and Stage III or Stage IV Cervical Cancer Undergoing Surgery to Remove Pelvic and Abdominal Lymph Nodes	Studying samples of tumor tissue and blood from patients to identify cancer biomarkers; the current primary objectives of this study are to detect the presence of T-synthase or COSMIC. Measuring the level of staining for Tn and STn antigens as well as measuring the differences in expression of 50 different genes on a customized glycogen array and differences in 10 carbohydrate structures using a customized glycan array.	Study is ongoing but not recruiting	NCT00460356

The Association between Alpha 1 Acid Glycoprotein Level and Outcome Metastatic Cancer Treated with Docetaxel	The association between the baseline plasma level of alpha 1 acid glycoprotein and progression-free survival of docetaxel based therapies in patients with metastatic nonsmall cell lung carcinoma, breast cancer, gastric cancer, prostate cancer, and bladder cancer.	Study is not yet open for participant recruitment	NCT00897962

Blood Glycan Biomarkers in Women with Stage IV Breast Cancer	Profiling serum glycan biomarkers in patients with metastatic breast cancer, healthy controls, and patients with noncancer medical illness.	Study is active but no longer recruiting	NCT00897962

Glycan Analysis in Diagnosing Cancer in Women with Ovarian Epithelial Cancer and in Healthy Female Analysis	Comparison of a new assay to the standard CA125 assay.	Study is currently recruiting participants	NCT00628654
